# The Mississippi Dental Association

**Published:** 1896-07

**Authors:** Jerome M. Walker


### PROCEEDINGS.

### The Mississippi Dental Association.

Reported for the DENTAL REGISTER by JEROME M. WALKER.

The Mississippi Dental Association held its annual session for 1896 in Jackson, April 21–23.

The President, Dr. T. C. West, of Natchez, occupied the chair.

The President, in his annual address, suggested a radical change in the working methods of the association. Instead of relying upon the chairmen of the different sections to prepare essays, he suggested the appointment of a committee, annually, to select subjects for the papers and discussions of the next annual meeting, and also to select the essayists and some one to open the discussion of each subject. The subjects being announced in advance, the members will be better prepared for intelligent discussion.

The suggestion having been adopted, at a later session the following list of topics was announced:

(1) Education.
(2) Use and Abuse of Plastics.
(3) Development of the Mandible and Maxilla.
(4) Metallurgy.
(5) Prosthesis.

The President suggested that discussion of the clinics also be made a special order of business, as calculated to add materially to the benefit to be derived from the clinics and in adding to the interest of the meeting.

This suggestion was also adopted, and was found to be especially interesting. The clinics were of unusual value, prominent operators from other States having been secured as clinicians.

PROF J. A. DALE, of Vanderbilt Dental Department, gave clinics in crown and bridge-work, the work being rapidly and skillfully executed. He also gave clinics in Orthodontiæ, by the Jackson method, exhibiting and constructing the "crib" appliances.

PROF. T. P. HINMAN, of the Atlanta Dental College, gave a clinic in bleaching teeth with pyrozone, 25 percent, a new and original feature being the application, after the tooth is bleached to normal color, of a clear, chloroform solution of gum mastic to the walls of the cavity and root-canals, the evaporation of the chloroform leaving a clear, transparent layer against the cavity-walls, and entering the open tubuli or canals, preventing future discoloration. Dr. T. P. Hinman also gave a very interesting and instructive "chalk-talk" on the subject of bleaching teeth.

He drew on the blackboard an illustration of a central incisor, supposed to be dead and discolored. Indicattng the usual point of opening into the pulp-chamber from the palatine surface, he said: "You are all, of course, very careful to remove all the decomposed tissue from the pulp-chamber and from the root-canal, but do you always remember these pockets (indicating the horns of the pulp), dipping down toward the cutting edge of the tooth? Unless they are thoroughly cleansed and filled, discoloration will return in spite of all your success in bleaching the tooth. Before undertaking to bleach the tooth, after all decomposed tissue has been removed, the root-canal must be well filled a certain distance down from the apex, preferably with oxide of zinc and aristol (equal parts), and a little oil of cassia. The tooth is then to be bleached with pyrozone, heating and drying the tooth between each application. The 25-percent ethereal solution is used, applied on a pellet of cotton to saturation, the surrounding tissues being protected by the rubber dam. The cavity is temporarily filled with gutta-percha, and, if necessary, the pyrozene application repeated the next day. When the tubuli have thus been bleached, and the tooth dehydrated, a chloroform solution of clear, transparent gum-mastic is applied to the walls of the cavity and the unfilled portion of the root-canal. The open tubuli take it up, and it hardens immediately, forming a white, transparent cavity-lining, and preventing any subsequent discoloration. Cement is then inserted against the cavity-walls, and the cavity filled as described."

Dr. Hinman has been forced to the conclusion that pyrozone 3 percent (medicinal), used as a mouth-wash, is very deleterious to amalgam fillings. He has observed fillings that had been doing good service for ten or fifteen years simply ruined by the chemical action of the pyrozene upon them.

The 25 percent is a powerful escharotic, the glyceride of tannin gives prompt relief in case of burn.

Prof. Frank Holland, of the Southern Medical College, Atlanta, Ga., Dental Department, did some very extensive and beautiful gold contour-restoration work, using Perry's Separators and the Electric Mallet, restoring badly decayed first and second bicuspids, and first molar (distal, morsal, and mesial), at one sitting, with great skill, rapidity and apparent ease. Prof. Holland also extracted a badly-impacted third molar with great skill.

Mr. Esterly, of the Atlanta branch house of the S. S. White Dental Manufacturing Company, demonstrated the Hollingsworth system of crown and bridge-work, constructing a number of crowns, and showing some novel and interesting features in the adaptation of the Hollingsworth system.

At the last annual meeting of the association a committee was appointed to secure the enactment of amendments to the dental law.

The Chairman of the Legislative Committee, Dr. J. H. Magruder, of Jackson, reported that a bill, embodying the amendments to the dental law, approved by the association at its last annual meeting, had been introduced in the Senate and passed without a dissenting vote, was introduced in the House, where, with some minor amendments, it passed almost unanimously. As amended it again passed the Senate unanimously; but notwithstanding this remarkable unanimity of sentiment on the part of the representatives of the people of the State, it was returned by the Governor without his signature, on the ground that it was "class legislation," and that it "gave the association too much power." The "power" given to the association by the proposed amendment lies in limiting the Governor, in the appointment of the Board of Dental Examiners, to choice from a list of *fourteen* chosen for recommendation by the association.

By the present law, a full new Board is appointed by each Governor, as soon as possible after his election, the entire Board serving during the full term of office of the Governor appointing it. One of the proposed amendments was that one member should go out every year, his place to be filled by the Governor by appointment from the list of fourteen mentioned, thus providing for one member of four years' experience always on the Board. The committee was continued, with instructions to interview the Governor and lay before him the methods adopted in other States, and to so modify the bill as to secure the Governor's consent to having it brought up before the next Legislature.

Dr. A. B. Kelly, of Yazoo City, read a paper on "The Use in Dental Practice of (1) Arsenious Oxide, (2) Campho Phenique, and (3) Fluo-Silicate of Sodium." This paper was an interesting *resume* of the varied applications of these important medicinal agents, and brought out an extended discussion of the subject of pulp-devitalization and root-canal filling, in which Drs. W. T. Martin, T. C. West, J. P. Broadstreet, T. P. Hinman, L. Holland, W. E. Walker, Frank Smith, L. A. Smith, and J. A. Dale took part.

DR. HOLLAND applies oil of cloves to the pulp for fifteen minutes for applying arsenic, and usually accomplished the death of the pulp without any discomfort to the patient.

DR. T. P. HINMAN applies tricresol to the minute fibers of living pulp-tissue, often so difficult to remove. A heated broach is then used to char the tissue.

DR. MARTIN uses trichloracetic acid for the same purpose.

DR. HOLLAND uses only a heated Evans' root-canal drier, or an electric root-drier. The removal is effected very quickly and without pain.

DR. T. C. WEST uses a 50-percent solution of sulphuric acid, which stiffens the fibrils so that they are easily removed. He also anæsthetizes the entire pulp with chloride of ethyl, instead of devitalizing with arsenic, and often removes it painlessly by this method.

DR. FRANK SMITH fills root-canals with points composed of paraffine or wax, and about half as much iodoform. These are introduced in the canal and followed by the heated Evans' root-drier, causing the material to penetrate into the dentinal tubuli.

DR. WALKER uses dressing-seal, with creosote worked into it, working it on a warm metal slab with a warm spatula. When the creosote is thoroughly incorporated, it is made into rolls and introduced into the canal with cold instruments and followed by hot air.

DR. HINMAN uses equal parts of oxide of zinc and aristol, with a little oil cassia, followed by a gutta-percha point.

DR. J. P. BROADSTREET, of Grenada, read a paper entitled "A Needed Educational Reform," being an able and eloquent plea for the education of the people in dental hygiene through the children in the public schools.

The paper was discussed and different methods suggested of accomplishing the much desired end. No definite conclusion was reached as to the best means of overcoming the many difficulties encountered in trying to reach those who are never met until driven to the dental chair by unbearable suffering.

Dr. Walker suggested the appointment of a committee to confer with the Superintendent of Education, with a view to the possible appointment of a lecturer on dental hygiene, to make the rounds of the public schools of the State, the lecture to be approved by the association, the lecturer to be indorsed by the State Superintendent of Education.

Dr. Holland does not think it would be possible to get the common-school children interested in a lecture on dental hygiene; they would look upon it as only an additional task imposed upon them.

Dr. West, on the other hand, thinks that even the youngest children could be interested in the subject, and spoke of the effect produced upon little kindergarten children by what "teacher says"—if "teacher says" they must do this, and must not do that, it seems to impress them more deeply than the routine of home-teaching.

The subject of the Dental Protective Association was brought before the association for discussion by Dr. Walker, as a matter in which every member of the dental profession was individually interested. Drs. Luckie and Martin seconded the remarks of Dr. Walker, and considerable interest was manifested in the matter. The subject of the proposition made to the dental profession by the management of the Army Medical and Surgical Museum, at Washington, was presented by Dr. J. A. Dale, in a letter from Dr. Henry W. Morgan, and the president was authorized to appoint a committee to secure contributions from the Mississippi Dental Association.

An invitation to hold a joint meeting with the State Societies of Alabama, Georgia and Florida was received. While appreciated, the invitation was necessarily declined, the charter of the association requiring that the annual meetings be held within the borders of the State, the "legal domicil" being the State capital, Jackson.

The election of officers resulted in the choice of Dr. Frank H. Smith, Water Valley, President; Dr. P. H. Wright, Senatobia,

Vice-President; Dr. J. P. Broadstreet, Grenada, Secretary; Dr. C. C. Crowder, Kosciusko, Treasurer.

The association adjourned to meet on the Wednesday of the week in which occurs the next meeting of the Board of Dental Examiners, the date of which is uncertain until the fate of the proposed amendments to the dental law is finally decided.

